# 1D Titanium Dioxide: Achievements in Chemical Sensing

**DOI:** 10.3390/ma13132974

**Published:** 2020-07-03

**Authors:** Navpreet Kaur, Mandeep Singh, Abderrahim Moumen, Giorgio Duina, Elisabetta Comini

**Affiliations:** SENSOR Laboratory, Department of Information Engineering, University of Brescia, Via Valotti 9, 25133 Brescia, Italy; n.kaur001@unibs.it (N.K.); mandeep.singh@unibs.it (M.S.); a.moumen@unibs.it (A.M.); giorgio.duina@unibs.it (G.D.)

**Keywords:** titanium dioxide, nanostructures, heterostructures, chemical sensing

## Abstract

For the last two decades, titanium dioxide (TiO_2_) has received wide attention in several areas such as in medicine, sensor technology and solar cell industries. TiO_2_-based gas sensors have attracted significant attention in past decades due to their excellent physical/chemical properties, low cost and high abundance on Earth. In recent years, more and more efforts have been invested for the further improvement in sensing properties of TiO_2_ by implementing new strategies such as growth of TiO_2_ in different morphologies. Indeed, in the last five to seven years, 1D nanostructures and heterostructures of TiO_2_ have been synthesized using different growth techniques and integrated in chemical/gas sensing. Thus, in this review article, we briefly summarize the most important contributions by different researchers within the last five to seven years in fabrication of 1D nanostructures of TiO_2_-based chemical/gas sensors and the different strategies applied for the improvements of their performances. Moreover, the crystal structure of TiO_2_, different fabrication techniques used for the growth of TiO_2_-based 1D nanostructures, their chemical sensing mechanism and sensing performances towards reducing and oxidizing gases have been discussed in detail.

## 1. Introduction

With the worldwide industrial and technological growth, there is a continuous demand for portable, low-cost and efficient sensing devices that can be used for various purposes such as environmental monitoring and health care. Due to this, researchers have made continuous efforts to find new materials or to grow existing materials in different forms such as thin films and nanostructures. In the field of chemical sensors, nanostructured metal oxides (MOX) are the most versatile and widely studied active sensing materials due to their unique physical/chemical properties [1]. Their wide band gap, along with the exceptional electrical properties, makes MOXs among the leading candidate for transparent electronic devices [2]. Indeed, tin oxide (SnO_2_) in different nanostructures forms such as nanowires [3,4], nanobelt [5,6] and nanorods [7,8] is the most widely investigated material in the field of chemical/gas sensing. 

Among the different nanostructures’ forms, one-dimensional (1D) nanostructures such as nanowires exhibit unique properties such as high surface to volume ratio, high crystallinity and controlled electrical properties which make them interesting for chemical sensing applications [3,9,10,11,12]. Because there is no requirement of preliminary gas diffusion to the surface, 1D MOX gas sensors showed faster response dynamics. Moreover, the self-heating phenomenon of nanowires can be used to reduce the power consumption of temperature-driven devices such as conductometric gas sensors, as nanowires attain high temperature when they undergo electrical resistance measurements, even when low electrical power has been used during the measurements [1,13].

Recently, MOXs such as zinc oxide (ZnO) [14], nickel oxide (NiO) [15], titanium dioxide (TiO_2_) [12] and tungsten oxide (WO_3_) [11] have been grown in the form of 1D nanostructures and were used for the detection of different gas analytes. Among these MOXs, TiO_2_ is one of the most versatile ones and is widely used for many different applications, including photocatalysis [16], sensors [17,18] and photovoltaic applications [19]. In particular, due to its exceptional physical/chemical properties, TiO^2^ was extensively used for different sensing applications such as chemical/gas sensors [20,21], biosensors [17,22] and chemical oxygen demand (COD) [23,24] sensors [25]. It typically exhibits n-type conductivity and, in nanostructured form, is chemically stable, biocompatible, biodegradable and inexpensive [25]. It exhibits three different crystal structures—namely, anatase, rutile and brookite, each of them possessing unique structural, electrical and optical properties [26]. Rutile TiO_2_ is widely used as a pigment, opacifier, isolator, and in switches, etc. due to its light-scattering ability, high refractive index and ultraviolet (UV) absorptivity, while anatase TiO_2_ is preferred for photovoltaic and photocatalytic applications [27]. On the other hand, brookite is the rarest occurring phase of TiO_2_, which is formed under a particular set of conditions. Due to this, brookite TiO_2_ has been very rarely investigated materials and its applications in fields such as photovoltaic applications [28] and lithium ion batteries [29] were recently discovered.

In this review article, recent achievements of 1D TiO_2_ chemical/gas sensors will be presented. Specifically, crystal structure of TiO_2_, MOX gas sensing mechanism and techniques used to grow 1D TiO_2_ nanostructures will be discussed in detail. In order to describe the recent achievements of 1D TiO_2_ chemical sensors, literature reported over the span of the last five to seven years will be presented.

## 2. Crystal Structure of TiO_2_

The structural properties of the active sensing material in a gas sensor (either in bulk, thin films or nanostructures form) are among the most important characteristics, as they influence the electrical, optical and mechanical behavior of the material. In particular, the surface structure and defectivity/stoichiometry of metal oxides has a great influence on local surface chemistry [26] and, consequently, they affect their performances as active sensing materials decisively. It is therefore important to understand the crystal structure of a chemo-resistive MOX to be used in a sensing device and to study how its structural properties may change as a function of the growth parameters (such as temperature). Hence, in this section, we will discuss the crystal structure of TiO_2_.

TiO_2_ exists in nature as well-known minerals rutile (tetragonal), anatase (tetragonal) and brookite (orthorhombic) [30]. In addition to these forms, two high-pressure forms of TiO_2_ also exist, i.e., a monoclinic baddeleyite-like form and an orthorhombic α-PbO_2_-like form, which were found at Ries Crater in Bavaria [31]. Indeed, rutile is the most thermodynamically stable polymorph of TiO_2._

**Figure 1:**
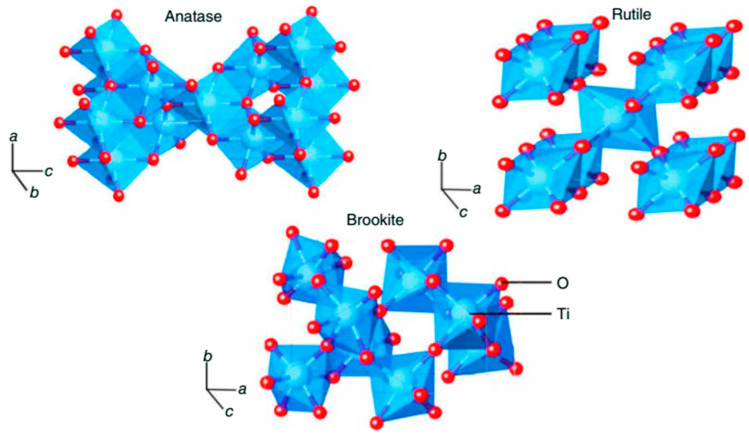
Different crystal structures of titanium dioxide (anatase, rutile and brookite). Reprinted with permission from ref. [32] WILEY Copyright 2019.

Figure 1 shows the crystal structures of the TiO_2_ anatase, rutile and brookite phases [32], and their corresponding crystal data are shown in Table 1 [26,33,34]. It should be noted that, due to the rare existence of brookite phase, the anatase and rutile forms of TiO_2_ are the most exploited in real applications. 

All the crystal structures of TiO_2_ are made up of distorted octahedra, each one representing a TiO_6_ unit, where Ti^4+^ is at the center of the unit and coordinates six O^2−^ ions. The way in which octahedra assemble to form the TiO_6_ chain is different for each crystal structure. Anatase and rutile crystal structures are assembled by connecting the octahedra by their vertices and edges, respectively, while in brookite, both vertices and edges are connected [34].

**Figure 2:**
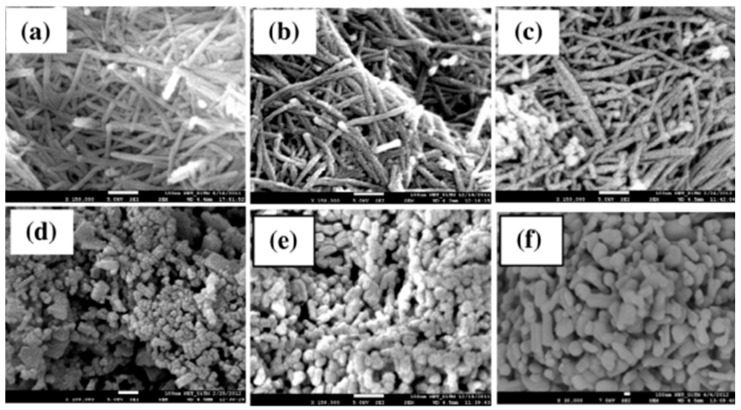
FESEM images of the as-prepared nanowires (**a**) and nanowires annealed at (**b**) 400 °C, (**c**) 500 °C, (**d**) 600 °C, (**e**) 700 °C and (**f**) 900 °C. Reprinted with permission from Ref. [12] Elsevier Copyright 2013.

At room temperature, the anatase phase is kinetically stable and does not undergo any phase transformation. Nevertheless, it can be converted into rutile via heating at high temperature [26]. The anatase-to-rutile thermally-induced transition in bulk TiO_2_ starts occurring at temperature T > 600 °C. M.N. Asiah et al. [12] have studied the phase transformation of TiO_2_ nanowires grown by means of a hydrothermal method. They found that, up to 500 °C, the nanowires (anatase phase) retained their morphology, and they started to change into smaller particles at around 600 °C. At 900 °C, the complete transformation from anatase to rutile phase occurred and the nanowires’ shapes changed to rod-like structures. Figure 2 shows the SEM images on TiO_2_ nanowires corresponding to the different temperatures [8]. 

Moreover, it was also found that the surface defects and lattice concentration play a crucial role during the phase transformation [34]. Specifically, the surface defects acted as nucleation sites and the rate of rutile transformation increased with an increase in surface defects. The removal of oxygen ions from TiO_2_ lattice (i.e., the formation of oxygen vacancies) also accelerated the rutile phase formation.

## 3. Synthesis of TiO_2_-Based 1D Nanostructures

This thematic study acquaints the fabrication techniques commonly used for the synthesis of TiO_2_ nanostructures. In the past decade, diverse fabrication methods, including chemical vapor deposition (CVD) [35], atomic layer deposition (ALD) [36], electrochemical deposition [37], hydrothermal methods [38], sol-gel [39] and electrospinning [40] have been successfully used for the preparation of high-quality TiO_2_ based nanostructures. The goal of this section is to elucidate the fabrication methods most commonly employed in the last five to seven years. 

### 3.1. Hydrothermal Synthesis

**Figure 3:**
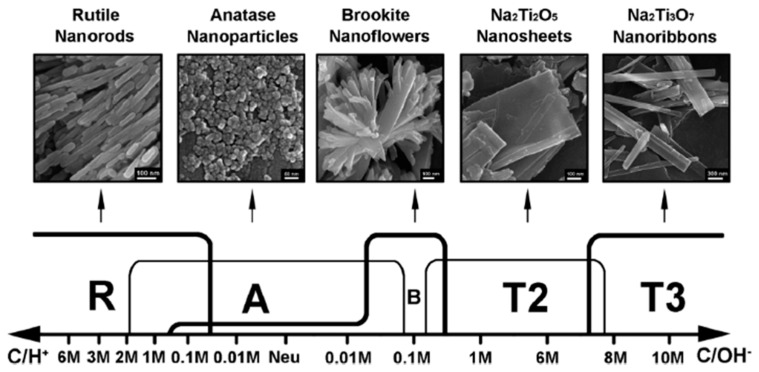
The effect of acid/alkali concentration. Rutile TiO_2_, anatase TiO_2_, brookite TiO_2_, dititanate Na_2_Ti_2_O_5_ and trititanate Na_2_Ti_3_O_7_ are represented by “R, A, B, T2 and T3”, respectively. Reprinted with permission from Ref. [38] RSC Copyright 2012.

Hydrothermal synthesis is one of the most widely used techniques for the growth of TiO_2_ nanostructures. This synthesis process is normally performed in steel autoclaves with or without Teflon liners under controlled pressure and temperature in an aqueous solution. All the experimental parameters involved in a hydrothermal process (e.g., annealing treatment, specific titanium precursor and process duration) influence the nanostructure growth. However, the nature of aqueous solution (acid/alkali concentration) has a major role in the structure morphology and crystallinity. B. Zaho et al. [38] reported that five different Titania/Titanate phases could be generated by employing a proper composition of acid (HCl) and alkali. High HCl concentration (6 M) lead to the formation of rutile nanorods, while monoclinic trititanate Na_2_Ti_3_O_7_ nanoribbons were formed at high NaOH concentration (10 M), as shown in Figure 3. These transitions have been explained by the Ostwald’s ripening mechanism [41]. 

**Figure 4:**
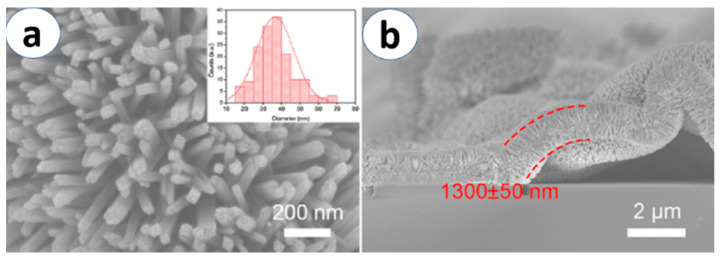
SEM images of TiO_2_ nanorods prepared using the hydrothermal method. (**a**) Top and (**b**) cross-sectional views of the nanorods. Reprinted with permission from Ref. [44] ACS Copyright 2020.

On the other hand, recently, many reports have been published on using hydrothermal synthesis for the growth of TiO_2_ nanowires (NWs) and nanorods (NRs) of rutile and anatase phase [42,43,44]. Generally speaking, typical processes to produce TiO_2_ nanorod arrays inside a steel autoclave involve a Ti precursor, HCl solution and deionized water, heated up at 150 °C for at least 9 h, followed by thermal annealing [42,43]. Another interesting work by G. Zaho et al. [44] reports about the growth of bridge-like structures of TiO_2_ NRs using isopropyl titanate as a precursor (Figure 4). Additionally, using Ti foil or TiO_2_ nanoparticles in appropriate concentrations of NaOH/ HCl and deionized water leads to successful growth of anatase TiO_2_ nanowires and nanotubes [45,46]. 

### 3.2. Electrochemical Anodization

Electrochemical anodization is an efficient method for the growth of well-aligned and highly ordered nanotubes (NTs). In a typical process, the NTs are produced at room temperature using a current generator in a cell consisting of an electrolyte and two electrodes—titanium (anode) and platinum (cathode). From the reported literature, it has been seen that the water-based ethylene glycol and ammonium fluoride aqueous electrolytes are the most commonly used for anodization of titanium to fabricate TiO_2_ NTs [47,48,49,50].

**Figure 5:**
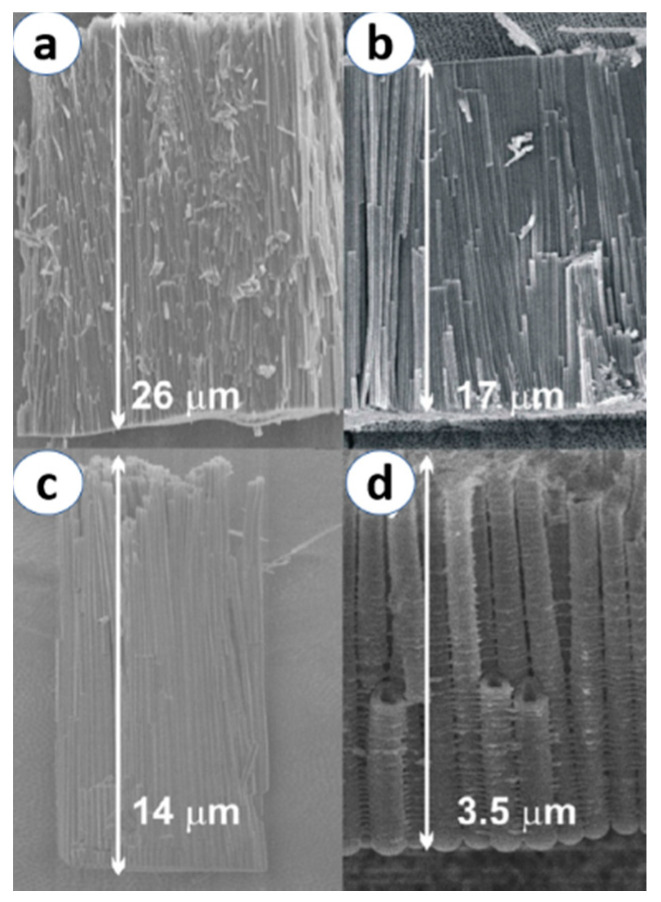
Cross-sectional images of TiO_2_ nanotubes synthesized by applying 60 V for 6 h using different electrolyte ages: (**a**) fresh electrolyte, (**b**) 6 h, (**c**) 25 h and (**d**) 50 h. Reprinted with permission from Ref. [51] Copyright 2015 Elsevier.

Generally, for the production of highly ordered NTs, an annealing treatment is required after the anodization. Different parameters such as voltage applied, anodization time and type of electrolyte solution are the major factors that influence the growth process [31,51,52]. It has been reported that highly uniform and longer NTs can be achieved when using high voltage, while the anodization time can majorly affect the length of NTs, but not their morphology [53,54]. Reports by B. Munirathinam et al. [53] and H. Sopha et al. [51] stated that the nature and the age of the electrolyte have major effects on the morphology and aspect ratio of NTs, as shown in Figure 5. This technique is usually carried out at room temperature, and it opens up great possibilities in the use of different substrates for an ultimate objective of fabricating new generation devices. 

### 3.3. Electrospinning

**Figure 6:**
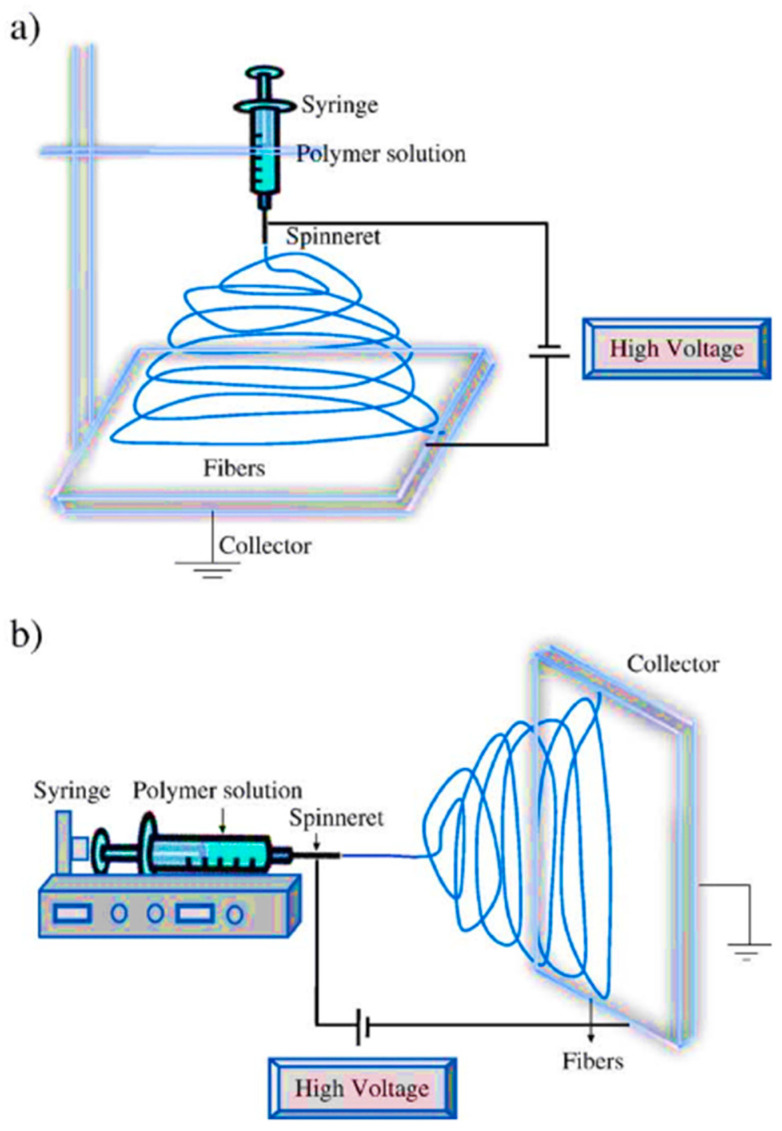
Schematic diagram of the electrospinning equipment (**a**) vertical and (**b**) horizontal setups. Reprinted with permission from Ref. [55], Copyright 2013 Elsevier.

In comparison to the above-mentioned growth techniques, electrospinning is one of the most simple and flexible methods to synthesize the large-scale 1D TiO_2_ nanostructures, especially long nanofibers of TiO_2_. Figure 6 shows a schematic diagram of the electrospinning equipment [55]. In this method, a liquid precursor solution is injected through a spinneret under an applied electric field and spin force to create fibrous structures. A post-annealing treatment is commonly needed to remove the solvent and to solidify the nanofibers. In recent years, electrospinning technique has been used not only to fabricate pristine nanostructures of TiO_2_, but also the composite [56,57,58,59,60]. 

**Figure 7:**
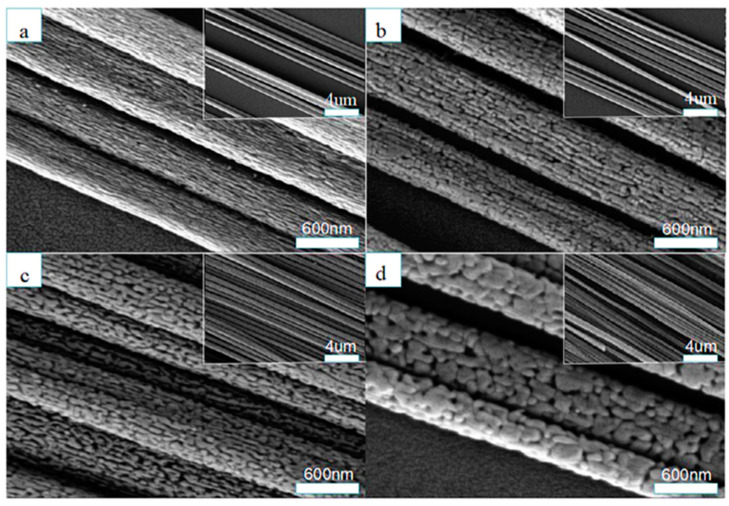
SEM images of aligned TiO_2_ NWs calcinated at different temperatures: (**a**) 500 °C, (**b**) 600 °C, (**c**) 700 °C and (**d**) 800 °C. Reprinted with permission from Ref. [56], Copyright 2019 Elsevier.

In a recent report by M. Zhou et al. [56], the authors synthesized TiO_2_ nanowires of different phases using the typical electrospinning method. Authors studied the effect of calcination temperature after the electrospinning of the nanowires at 500 °C, 600 °C, 700 °C and 800 °C for 2 h in air (shown in Figure 7). It was discovered that by increasing the annealing temperature, the grain size and pore size increase as well, and a phase transition from rutile to anatase TiO_2_ occurs, while the band gap decreases. 

Another interesting report, authored by S. Wang et al. [57], reported the fabrication of TiO_2_ nanowires with two different methods, namely, an ethylene glycol-mediated method and a high-voltage electrospinning method. The nanowires synthesized via the solution method were found to be relatively uniform, having an average diameter around 550 ± 90 nm and a length of 26 ± 5 μm. On the other hand, nanowires grown with the electrospinning method were found to be much smaller, exhibiting an average diameter of 70 ± 5 nm and a length of 12 ± 5.5 μm. In 2017, F. Li et al. [59] presented the coaxial electrospinning method using two precursors to fabricate the core-shell nanostructures of TiO_2_/SnO_2_. The structural characterizations showed two mixed phases of tetragonal SnO_2_ and rutile TiO_2_ with no other impurity diffraction peaks evidenced by X-ray diffraction analysis.

Moreover, besides the above-mentioned growth process for TiO_2_ nanostructures, the other least used techniques within the last five year were thermal oxidation [61], acid vapor deposition (AVO) [62] and the vapor phase growth process [63]. 

Finally, the various reports published in the last five years on the growth of TiO_2_ nanostructures and its composites have been collected in Table 2, while in Table 3, some the major advantages and disadvantages related to these techniques have been presented.

## 4. Working Principles of TiO_2_-Based Chemical Sensors

**Figure 8:**
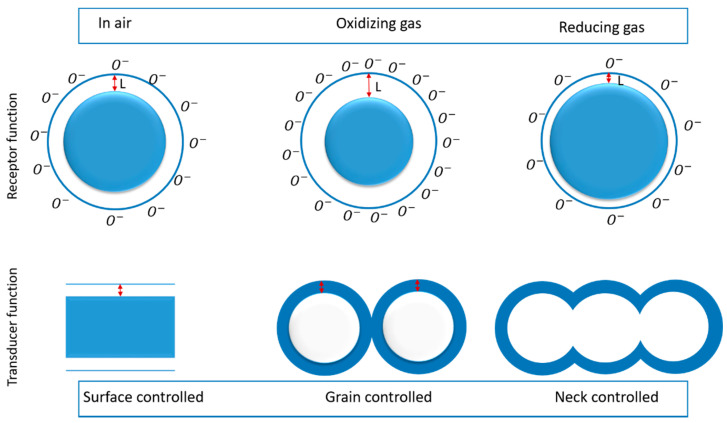
Schematic representation of gas sensing at different modes.

Most semiconductor-based gas sensors rely on the chemo-resistive effect—that is, the change of the electrical conductance or resistance upon the exposure to chemical compounds. In particular, the semiconductor sensing element resistance can increase or decrease when reacting with oxidizing (O_3_, CO_2_ and NO_2_) or reducing (H_2_, H_2_S, NH_3_ and VOCs) gases [66,67,71,75,76]. The sensing mechanism of TiO_2_-based chemical sensors is frequently explained by two processes: receptor and transduction function, as shown in Figure 8. 

The receptor process takes place on the surface of TiO_2_, comprising physisorption and chemisorption processes [77,78]. As TiO_2_ surface is exposed to air, the oxygen molecules start to physically adsorb on its surface. This process is determined by dipole and van der Waals interactions. Furthermore, these adsorbed molecules initiate capturing electrons from the conduction band of TiO_2_, while oxygen species chemisorb ($O^{-}$, $O_{2}^{-}$) on the surface. This chemisorption of oxygen ions creates a depletion region near the TiO_2_ surface. 

The receptor process efficiency is majorly dependent on physisorption and chemisorption processes, which in turn depend upon the sensor operating temperature. Specifically, at elevated temperature (100–500 °C), the chemisorption of oxygen ions occurs on the oxide surface and can be explained by the following equations [79]:

(1) $$O_{2\left(\mathrm{gas}\right)}↔O_{2\left(\mathrm{ads}\right)}$$

(2) $$O_{2\left(\mathrm{ads}\right)}+e^{-}↔O_{2\left(\mathrm{ads}\right)}^{-}\left(<100\;{}^{\circ}C\right)$$

(3) $$O_{2\left(\mathrm{ads}\right)}^{-}+e^{-}↔2O_{\left(\mathrm{ads}\right)}^{-}\left(100-300\;{}^{\circ}C\right)\;$$

(4) $$O_{\left(\mathrm{ads}\right)}^{-}+e^{-}↔O_{\left(\mathrm{ads}\right)}^{2-}\left(>300\;{}^{\circ}C\right)$$

Hence, the type of chemisorbed oxygen ions depends on the sensors’ operating temperature and determines the reaction mechanism with the gas analyte. Due to this, metal oxides gas sensors are operated at different temperatures to find the optimal working conditions for each gas analyte. 

Secondly, the transducer process is determined by the transportation of electrons in TiO_2_ and their transformation into a readable electronic signal. This process is mainly divided into three different modes (shown in Figure 8), i.e., surface-controlled mode, grain-controlled mode and neck-controlled mode [77,78,80,81]. (i) The surface-controlled model is mainly related to compact thin film structures, in which the gases react to the surface of TiO_2_ rather than the bulk. (ii) On the other hand, in the grain-neck-controlled model, the gases reactivity is much higher. This is due to the fact that in the granular structures with higher porosity, each grain and neck boundary will create a depletion region that leads to an increase in the sensor resistance, thus improving the sensitivity of the TiO_2_ nanostructures.

Furthermore, the surface to volume ratio, grain size and porosity of the fabricated TiO_2_ nanostructures can majorly affect the above mention process. In this direction, in the last decade, different 1D nanostructures of TiO_2_ and their heterostructures, as well as element doping of TiO_2_, have been highly studied in chemical sensing applications [58,61,67,71,74].

## 5. Chemical Sensing Properties

### 5.1. Sensing of Reducing Gases

Among many metal oxides, 1D TiO_2_ nanostructures have been extensively used as sensing materials to build a high-performance chemical sensor for the detection of reducing gases such as H_2_, VOCs (ethanol, acetone etc.) H_2_S, NH_3_ and so on [40,71,72,78].

Among chemical compounds crucial to detect and monitor, H_2_ is one of the most important. H_2_ is used in applications such as fuel cells [82]; it is extremely flammable and explosive, and, therefore, its detection is crucial to guarantee a safe exploitation of H_2_ based energy-related systems. M. Enachi et al. [67] reported the sensing performances of three different phases of TiO_2_ nanotubes including amorphous, anatase and mixed anatase/rutile. The phase transition was achieved using thermal treatment by annealing the sample after the deposition procedure. The authors separated a single nanotube and transferred it to the chip containing Au metal contacts to study the suitability of an individual TiO_2_ nanotube as a hydrogen sensor and to investigate the sensing performance for the three obtained phases. The sensing device based on single nanotubes with mixed anatase/rutile phase exhibited a faster response and recovery time of ~0.7 s and ~0.9 s, respectively, and the response (defined as $\frac{I_{H_{2}}}{I_{\mathrm{air}}}$) was about 3.5 at room temperature. 

In order to improve the sensing performances of 1D TiO_2_, many strategies have been proposed in the literature, such as increasing the response and reducing the power consumption using photoactivation, improving the selectivity, enhancing the response and reducing the working temperature by functionalization TiO_2_ with transition metals, and maximizing the sensitivity by bulk doping and by building heterojunctions.

A. Nikfarjam et al. proposed photoactivation as a method to increase the response of the thermoactivated TiO_2_ nanofibers network [40]. The best response was found 18 and 96 towards 25 and 200 ppm of H_2_, respectively, under UV illumination at 190 °C, significantly higher compared to 1.8 and 10.1 in dark at 290 °C. The sensor exhibited a fast response, and a long-term stability of 9 months was reported. Moreover, the optimal working temperature decreased from 290 to 190 °C. This increase in performance was attributed to the reduction of the activation energy between H_2_ molecules and TiO_2_ surface under UV irradiation, and to the increase in oxygen chemisorption (O^-^) density for its interaction with H_2_, even at low temperatures. Such results are interesting for the possibility to achieve battery-operated devices thanks to the reduction in power consumption and miniaturization of the gas sensor device.

**Figure 9:**
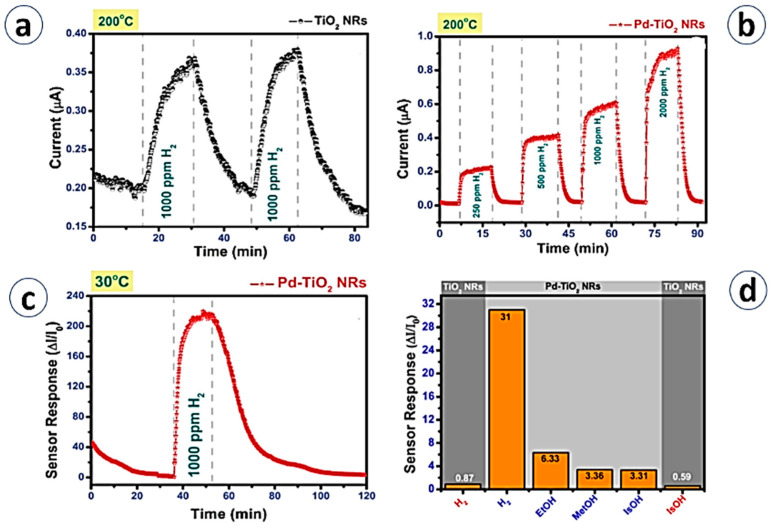
(**a**) Dynamic response of TiO_2_ nanorods at 200 °C. (**b**) Dynamic response of Pd-TiO_2_ at 200 °C toward different H_2_ concentrations. (**c**) Dynamic response of Pd-TiO_2_ at 30 °C toward 100 ppm of H_2_ (**d**) Sensor responses of TiO_2_ and Pd TiO_2_ nanorod device for H_2_ and VOCs at 200 °C. Reprinted with permission from Ref. [75] Copyright 2016 Elsevier.

Another way to increase sensing performances is the functionalization of the TiO_2_ surface. For example, TiO_2_ nanorods produced using the hydrothermal method were modified with Pd nanoparticles [75]. Figure 9 shows Pd/TiO_2_ NRs exhibiting a great enhancement toward 1000 ppm of H_2_ at 200 °C with a 35-fold higher response compared with TiO_2_ NTs due to the spillover effect. Specifically, Pd nanoparticles on the TiO_2_ NRs catalytically stimulate the dissociation of molecular oxygen and atomic products obtained during this process diffuse to the metal oxide. This process greatly enhances the oxygen vacancies on the TiO_2_ surface and directly affects the sensing performance. Furthermore, together with the good selectivity towards H_2_ over other VOCs, the working temperature was reduced from 200 to 30 °C and the response increased to 250, thanks to the hydrogen collector activity of Pd. 

Furthermore, the combination of two different catalysts may increase performance even more. Shoutian Ren et al. [45] achieved an enhancement in response and response time (350% and 42 s) for AuPd-TiO_2_ as compared with Pd-TiO_2_ (2% and 21 s) at room temperature. They also investigated the sensing mechanism by formation of a local heterostructure between Au and Pd, which enhanced electrocatalytic activity of alloyed macrostructures and increased the hydrogen dissociation process further; a crucial role was played by the size and the content of Pd/Au ratios. Moreover, heterostructures based on titanium oxide such as Co_3_O_4_/TiO_2_ and CuO/TiO_2_ also revealed high potentialities for hydrogen detection [48,49].

Other chemical species that deserve attention are volatile organic compounds (VOCs). VOCs, which commonly arise from painting, vehicle exhaust emissions and oil refining, are hazardous compounds. They have serious effects on air quality and human health. TiO_2_ nanostructures have been investigated as VOC sensors. In particular, TiO_2_ nanorods showed good sensing performances in triethylamine (TEA) detection [43]. The fast response time (2s), low detection limit (0.1 ppm) and good selectivity over acetone, ethanol, benzene, chlorobenzene, methanol and N-propanol make it a good choice for controlling fish and seafood freshness [43]. In this study, the sensing mechanism of TEA has been proved using XPS, confirming that TEA adsorbs on the surface of TiO_2_ NRs before reacting with pre-adsorbed oxygen. Ethanol and acetone are the most studied among VOCs targets for 1D TiO_2_ [83,84,85]. In this context, it has been shown that TiO_2_ nanorods with a diameter of 500 nm show a good response of 9, 13 and 20 towards 10, 300 and 1000 ppm of acetone at 500 °C with response and recovery time ranging from 11 to 14 s and 4 to 8 s, respectively, showing a fast acetone detection [86]. TiO_2_ nanotubes with 0.85 µm lengths and wall thickness of 13 nm show efficient low-temperature (150 °C) ethanol sensing with an 80% response exposed to 1000 ppm, and response and recovery times of 11 and 117 s, respectively [85].

**Figure 10:**
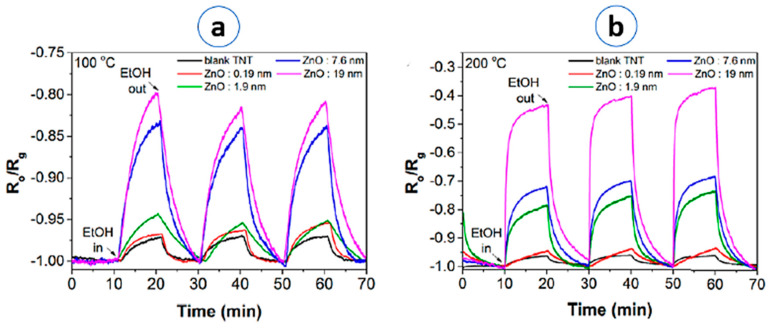
Ethanol sensing responses of TiO_2_ NTs and TiO_2_/ZnO (0.19, 1.9, 7.6 and 19 nm) core/shell (**a**) at 100 °C and (**b**) at 200 °C. Reprinted with permission from Ref. [47] Copyright 2018 WILEY.

However, in the last five years, greater interest was focused on heterostructured and composite titania material for the purpose of increasing their sensing performances. Siowwoon Ng et al. [47] investigated TiO_2_/ZnO core-shell nanostructures for the development of ethanol sensors. In their work, a fascinating strategy has been employed to control the ZnO shell thickness (0.19, 1.9, 7.6 and 19 nm). The response of TiO_2_/ZnO core shell nanostructures towards 1930 ppm of ethanol is improved by increasing the thickness of ZnO shell; with a 19 nm thickness, it is 11 times higher than the one of pristine TiO_2_ NTs at 200 °C (Figure 10). The sensor response dynamics are enhanced, and good repeatability and stability have been achieved. Similar results have been established on 1D TiO_2_ heterostructures with Nb_2_O_5_, Al_2_O_3_, V_2_O_5_ and TiO_2−x_ materials [58,61,74,87].

Enhancing sensing performances of heterojunction are due to band bending at the interface attributed to Fermi level alignment via charge transfer; furthermore, active sites are provided for gas adsorption and synergistic reaction effects [47]. The improvement of sensing characteristics toward acetone have been investigated using different strategies, either by using heterojunction structure as reported by Feng Li et al., [59] who studied TiO_2_/SnO_2_ core shell nanofibers and observed a response improvement from 3 to 14 for TiO_2_ and TiO_2_/SnO_2_, respectively, at 280 °C; by surface sensitization [71]; or sometimes by bulk doping [73].

1D TiO_2_ also showed good potentialities as NH_3_ and H_2_S chemical sensors. Capability of detection of low concentrations of NH_3_ at room temperature in different humidity environments has been proven [44]. The sensor exhibited good long-term stability (29 days), with a response of 102% toward 100 ppm of NH_3_. Moreover, a decrease in response was observed by increasing humidity levels from 28% to 75%. The study also showed a good selectivity to NH_3_ over CH_4_, H_2_S, NO_2_, H_2_, ethanol, methanol, acetone, diethylaniline (DEA) and TEA. 

Another important chemical compound in terms of health, security and safety is H_2_S. H_2_S is a colorless gas that is very poisonous and extremely flammable. Exposure to H_2_S concentration ranging from 20 to 100 ppm may cause eye burns and respiratory problems, and sometimes paralysis and even death [88,89]. Therefore, finding equipment for early detection of H_2_S is highly recommended. In this context, P. M. Perillo et al. fabricated flexible H_2_S sensors based on anatase TiO_2_ nanotubes [72]. The tube length was 12 µm, while the pore and wall diameters were approximately 100 nm and 30 nm, respectively. The flexible sensor showed response of 75% towards 25 ppm of H_2_S at room temperature with excellent long-term stability (6 months).

Table 4 reports a literature survey for the TiO_2_ nanostructure and heterostructure growth procedures and their chemical/gas sensing performances towards reducing gases. 

### 5.2. Sensing of Oxidizing Gases

Environmentally hazardous oxidizing compounds such as NO_2_, O_3_ and CO_2_, etc. are among the major concerns for the human life and health [11,90,91,92,93]. For example, NO_2_ is a typical air pollutant byproduct of combustion facilities, aircrafts and automobiles. According to the American standard air quality monitoring protocols for NO_2_, the concentration should be detectable in the range of 3−25 ppm. Moreover, the decomposition of NO_2_ by solar irradiation is a common source of ozone (O_3_) production. The safe concentration of ozone is 50 ppb for continuous exposure and 100 ppb for short-term exposure has been set for the developed countries. Thus, a low-cost, reliable sensor with high selectivity and sensitivity is of huge demand for environmental safety and industrial control purposes. In this context, TiO_2_ nanostructure-based sensors have been widely investigated for the detection of these hazardous pollutants [48,50,60,61,63,64,65,66,94]. 

A recent report by Z. Zhu et al. [66] presented highly sensitive NO_2_ sensors based on TiO_2_ nanowires. These hydrothermally grown nanowires-based TiO_2_ sensors showed very fast response and recovery time of 10 to 19 s respectively, with a response of 3.1 towards 100 ppm of NO_2_. Furthermore, TiO_2_ nanorods-based sensors have been widely used for the investigation of highly sensitive NO_2_ sensors. Z. Tshabalala et al. [64] presented a hydrothermal method followed by an annealing process to fabricated pristine TiO_2_ NRs. Different devices were prepared using different washing solutions such as distilled water and hydrochloric acid of different concentrations 0.25, 0.5 and 1 M, and were annealed at different temperatures afterward. Gas tests, performed at RT in dry air, show that the sensing properties depend on washing solution and annealing temperature. The best response (1300 at 40 ppm of NO_2_) was achieved by the samples cleaned with hydrochloric acid 1.0 M and annealed at 700 °C. The best optimized samples exhibit high porosity, which leads to the larger surface area, in turn increasing the response.

As discussed in pervious sections, in the last five years, much work has been done to achieve high sensing performance by doping of metal particles or by creating the oxide-to-oxide junction. D. Ponnuvelu et al. [94] reported on Au-decorated mixed phase TiO_2_ NRs fabricated by hydrothermal method for highly sensitive NO_2_ sensors. After Au particle decoration, not only did the response value increase, but also the working temperature decreased from 400 °C to 200 °C in comparison to bare TiO_2_ NRs. The sensor devices were tested toward very low concentrations of NO_2_ from 500 ppb to 5 ppm with a response of 135.5 to 15, respectively. The enhancement in NRs sensing performances is due to the catalytic effect of Au doping to the NRs surface that induces surface defects and the coexistence of mixed phases (metastable anatase and thermodynamically stable rutile) of TiO_2_. 

**Figure 11:**
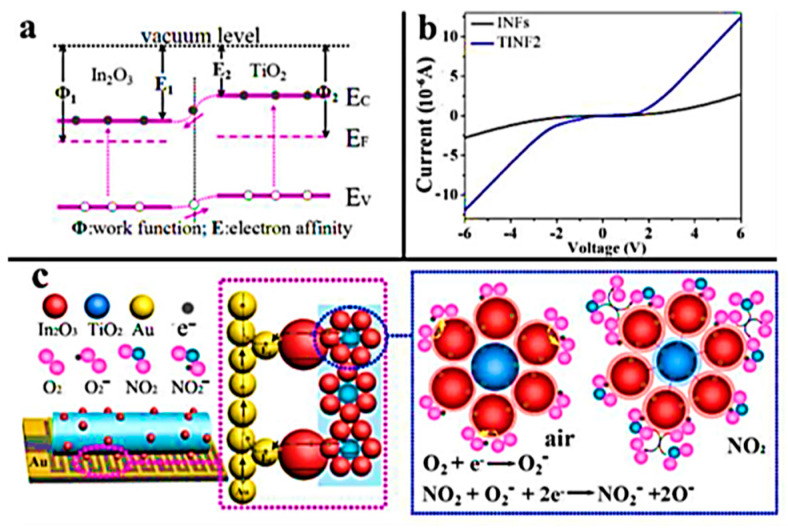
(**a**) Energy band diagram of In_2_O_3_ and TiO_2_, EC: conduction band and EV: valence band; (**b**) I-V curves measured for INFs and TINF_2_ thin film sensors in air at RT, which were treated at 110°C (the gate voltage Vg = 0.1); (**c**) The gas sensing reactions based on Schottky junction between Au electrode and In_2_O_3_ beads. Reprinted with permission from Ref. [46] Copyright 2015 American Chemical Society.

Constructing a junction between two similar or dissimilar materials is another successful method to improve the sensing performance of metal oxide-based devices [60,65]. L. Wang et al. [60] presented an interesting report on TiO_2_-In_2_O_3_ composite nanofibers (NFs) with In_2_O_3_ porous beads built using the electrospinning method for the detection of NO_2_. In particular, they developed a single-step process, followed by calcination, to achieve a nanoparticle–nanofiber structure. The porous single crystal NRs loaded on the composite NFs play a crucial role in the formation of Schottky junctions between the semiconductor materials and the gold electrodes. These junctions reduce the interfacial resistance when charge carriers flow through the contacts, thus increasing the overall conductance (shown in Figure 11). Moreover, the surface defects along with the NFs porosity give birth to a high surface-to-volume ratio which allows the adsorption of gases (O_2_ or NO_2_) and the electrons capture more effectively, even in the bulk. The resulting sensing devices exhibit superior performances at RT with a response of 95 toward 97 ppm of NO_2_.

Even though oxygen is not a toxic gas, the results reported by Wang et al. [95] from fabricating TiO_2_ NRs array using an acid vapor oxidation (AVO) technique are worth mentioning. The sensing tests were carried out in dry air at RT for O_2_ concentrations ranging from 1% to 16% (volume ratio). The responses were monotonically increasing with the concentration approaching 2.1 at 16%. At the same concentration, the best tradeoff between response and recovery time (55 s and 51 s) were obtained. These sensors have also demonstrated a good repeatability and selectivity against hydrogen and methane.

Table 5 comprises the literature survey for TiO_2_ nanostructures and heterostructures together with their growth techniques and sensing performance towards oxidizing gases.

### 5.3. Effect of Humidity

Generally, the response of the metal oxides gas sensor in the presence of humidity is reduced. The reduction in their gas sensing response in a wet environment occurs due to the competition of adsorption between the analyte and the water molecules. The moisture may absorb on the surface metal sites, reducing oxygen adsorption. In addition, hydrogen produced by the homolytic dissociation of water molecules subsequently reacts with the lattice oxygen of the metal oxides (MOX), forming surface hydroxyl species. Hence, in the presence of humidity, water is in competition with the absorption of hydrogen at the MOX surface. The reduction in the gas sensing response in a wet environment is commonly known as the “poisoning effect of water on metal oxide sensors” [96,97]. Moreover, at elevated temperatures, moisture may also be responsible for a restructuring of the surfaces, modifying the density of oxygen absorption sites.

## 6. Conclusions

Titanium oxide (TiO_2_) is a nontoxic and environmentally friendly material in nature with excellent biocompatibility and stability, which allows its nanomaterials to be adopted in a wide range of applications, especially in chemical/gas sensing. The 1D TiO_2_ nanostructures such as nanowires, nanotubes and nanofibers possess larger specific surface areas that contribute to the unique physical, chemical, optical and electronic properties of this material. TiO_2_-based 1D nanostructures have been grown using large-scale and high-yield techniques such as hydrothermal, electrochemical anodization and the electrospinning growth method showed exceptional sensing performances for the detection of different gas analytes such as VOCs, toxic and explosive gases. However, in the last five to seven years, great attention has been paid to improving the sensing performance of these nanostructures by doping of metal nanoparticle or by creating a junction of different MOX. These heterostructures show an enhancement in sensitivity, selectivity and lower operating temperature with faster responses and recovery times. The change in sensing performances of composite structures compared to pristine TiO_2_ is attributed to the heterojunction formation within the interface of two semiconducting materials, as well as catalytical and synergetic effects. Furthermore, the formation of nanostructured material also influences gas adsorption and diffusion, which leads to a change in sensing performances. 

Concluding, in the field of chemical/gas sensing, there has been much progress achieved in growth of various 1D TiO_2_ nanostructures and heterostructures using different fabrication techniques. The effect of different phases of TiO_2_ on its sensing properties has been widely investigated. In this review, we have presented the recent achievements in the field of TiO_2_-based 1D nanostructures fabrication and understanding of their sensing mechanism and their sensing performance related to different gas analytes.

## Figures and Tables

**Table 1:** Crystal structure data of the three main polymorphs of TiO_2_ [26,33,34].

Crystal Structure	System	Space Group	Lattice Constants (nm)			
			a	b	c	c/a
Rutile	Tetragonal	$D_{4h}^{14}$—P4_2_/mmm	0.4584	-	0.2953	0.644
Anatase	Tetragonal	$D_{4h}^{14}$—I4_1_/amd	0.3733	-	0.937	2.51
Brookite	Rhombohedral	$D_{2h}^{15}$—Pbca	0.5436	0.9166	-	0.944

**Table 2:** Summarization of different materials, parameters, crystal phase involved in the growth process of TiO_2_ nanostructures and its composites for different fabrication techniques.

Hydrothermal					
Metal Oxide	Materials/Parameters	Nanostructure Type	Sized	Crystalline Phases	Ref./Year
TiO_2_	Titanium butoxide, hydrochloric acid and deionized water/ Temp. = 150 °C Time = 18 h	Nanorods	length = 5 μm diameter = 100 nm	rutile	[42]/2015
TiO_2_	Isopropyl titanate, hydrochloric acid Temp. = 150 °C Time = 3, 6, and 9 h	Nanorods	length = 4200 nm diameter = 120 nm	rutile	[44]/2020
TiO_2_	TiCl_4_, HCl and deionized water Temp. = 180 °C Time = 3 h	Nanorods	diameter = 21.20 nm length = NA	anatase/rutile mixed phases	[64]/2017
TiO_2_–Ag_2_O	TiCl_4_, deionized water Temp. = 180 °C Time = 3 h	Composite Nanorods	diameter = 100 nm	rutile	[65]/2019
Bi-TiO_2_	[Bi(NO_3_)_3_·5H_2_O] and TiO_2_ nanoparticles with NaOH and water. Temp. = 140 °C Time = 24 h	Nanotubes	diameter = NA length = NA	anatase	[46]/2017
TiO_2_	Titanium (IV) isopropoxide, NaOH and ethanol. Temp. = 150 °C Time = 20 h	Nanowires	length = 1825 nm diameter = 30–50 nm	anatase	[66]/(2018)
**Electrochemical Anodization**					
TiO_2_	Ti foil in ethylene glycol, acide phosphorique (H_3_PO_4_) and hydrofluoric acid HF. Anodization = 120 V Temp. = RT Time = 2 h	Nanotubes	length = 20 µm diameter = 80–120 nm	anatase/rutile mixed phases	[67]/2015
7TiO_2_	Ti foil in ethylene glycol, deionized water and ammonium fluoride (NH_4_F) Anodization = 1 From 0 V to 60 V Time = 4 h Temp. = RT	Nanotubes	length = 50 μm diameter = 120 nm	anatase	[51]/2015
TiO_2_	Ti foil in NH_4_F and (NH_4_)_2_SO_4_ and deionized water Anodization = 20 V Time = 2 h Temp. = RT Thermal annealing at 750 °C for 4 h in air	Nanotubes	length = NA diameter = 50–80 nm	anatase	[68]/2020
p-Co_3_O_4_/n-TiO_2_	Ti foil, NH_4_F ethylene glycol, cobalt (II) nitrate hexahydrate and water Anodization = 50 V Time = 1 h Temp. = 500 °C	Nanotubes	length = NA diameter = 80 nm	anatase	[48]/2018
Pd decorated TiO_2_	Ti foil in NH_4_F and ethylene glycol. Anodization = 60 V Time = 45 min Temp. = RT Thermal annealing at 6 h at 500 °C in ambient air	Nanotubes	length = NA diameter = 40 nm	anatase	[69]/2016
PbS quantum dots/TiO_2_	Ti foil in ethylene glycol solution containing NH_4_F and H_2_O Pb(NO_3_)_2_ dissolved in methanol and Na_2_S in methanol. Anodization = 50 V Time = 5 h Temp. = RT	Nanotubes	length = 18 μm diameter = 80 nm	anatase	[70]/2016
Ni and Pd-modified TiO_2_	Ti foil, NH_4_F, ethylene glycol and water. NiCl_2_ and PdCl_2_ Anodization = 20 V Temp. = 300 C Time = 3 h	Nanotubes	diameter = 50–60 nm length = 470 nm	anatase	[71]/2015
TiO_2_	Ti foil and NH_4_F with H_2_O in ethylene glycol Anodization = 50 V Time = 3 h Temp. = RT	Nanotubes	diameter = 100 nm length = NA	anatase	[72]/2016
Co-doped TiO_2_	Ti foil in glycol solution with ammonium fluoride and deionized water. Anodization = 30 V Time 2 h Temp. = RT	Nanotubes	diameter = 110 nm length = NA	anatase	[73]/2019
**Electrospinning**					
TiO_2_	Titanium tetraisopropoxid (C_12_H_28_O_4_Ti), ethanol, acetic acid Stirring time = 15 min Voltage = 18 kV Rate = 2 mL/min Calcination temp. = 300 °C, 500 °C, 700 °C, 900 °C	Nanofibers	lengths = NA diameters = 50 nm, 80 nm, 130 nm, 200 nm	anatase/rutile mixed phase	[40]/2015
In_2_O_3_ beads @ TiO_2_-In_2_O_3_ composite	TBT (tetrabutyl titanate), indium nitrate hydrate, ethanol, DMF (dimethylformamide), PVP (polyvinylpyrrolidone) Stirring time = 6 h Voltage = 16.0 kV Rate = 0.25 mL·h^−1^	Nanofibers	lengths = tens of µm diameter = 150–250 nm	polycrystalline TiO_2_-In_2_O_3_ composite	[60]/(2015)
Nb_2_O_5_-TiO_2_	Titanium isopropoxide, polyvinylpyrrolidone, acetic acid, ethanol stirring time = 12 h Voltage = 18.0 kV, −4.0 kV Rate = 1.5 mL/h, Calcination temp. = 500 °C	Nanofibers	lengths = NA diameter = 121.3 nm	anatase/ rutile mixed phase	[58]/2019
TiO_2_/V_2_O_5_	Tetrabutyl titanate, poly-vinylpyrrolidone, ethanol, acetic acid stirred time = 20 min Ti/V molar ratio of 4:1 Annealing Temp = 450 °C	Nanofibers	length = µm range diameter = 60 nm	anatase/ rutile mixed phase	[74]/2016
TiO_2_-SnO_2_	Dimethylformamide, ethanol stirring time = 10 min SnCl_2_·2H_2_O stirring time = 8 h voltage = 18 kV	Nanofibers	N/A	rutile	[59]/2017

**Table 3:** Advantages and disadvantages of hydrothermal, electrochemical anodization and electrospinning techniques.

Hydrothermal		Electrochemical Anodization		Electrospinning	
Advantages	Disadvantages	Advantages	Disadvantages	Advantages	Disadvantages
Simple, easy and low-cost synthesis method	The reaction takes long time	Production of High quality of 1D nanostructures specially nanotubes	Mainly used for nanotubes growth	Ease to fabricated composites	Limited control of structure porosity
Production of High quality of 1D nanostructures specially nanorods	Utilization of highly concentrated NaOH solution	Ordered and aligned structure	The mass-produced is limited	High efficiency	Use of toxic solvents
The morphology is controlled by synthesis parameters	Difficult in achieving uniform size	high aspect ratio (length/diameter ratio)	Utilization of toxic electrolyte: Hydrofluoric acid HF	Process simplicity	-
Easy addition of additives for doping	length/diameter ratio is smaller than the ratio produced by electrochemical anodization	Growth at room temperature	high production cost	Mass production	-
-	-	Aspect ratio controlled by synthesis parameters	Difficulties in separation of film from substrate	-	-

**Table 4:** Summary of the chemical/gas sensing properties of TiO_2_-based nanostructures and heterostructures towards reducing gases.

Material	Synthesis Method	Target Gas/ Concentration	Response (S)	Temperature/ Humidity	Response/ Recovery Time	Ref./Year
TiO_2_ NTs	Electrochemical anodization	H_2_/100 ppm	S = (I_g_/I_a_) 3.5	RT/Dry air	0.7 s/0.9 s	[67]/2015
TiO_2_ NFs	Electrospinning	H_2_/50 ppm	S = (R_a_/R_g_) 30	190 °C with UV irradiation/Dry air	2s/6.9 s	[40]/2015
TiO_2_ NRs	Hydrothermal	H_2_/2000 ppm	S = (∆I/I × 100) 215%	200 °C/Dry air	NA	[42]/2015
p-Co_3_O_4_/n-TiO_2_ NTs	Electrochemical anodization	H_2_/1000 ppm	S = (∆I/I) 6	200 °C/50%	10 min/5 min	[48]/2018
Pd decorated TiO_2_ NTs	Electrochemical anodization	H_2_/10ppm	S = (∆R/R) 1.25	180 °C/in dry synthetic air	20 s/40 s	[69]/2016
PdAu decorated TiO_2_ NWs	Hydrothermal	H_2_/5 ppm	S = (∆I/I×100) 350%	RT/-	42 s/NA	[45]/2016
TiO_2_/ZnO core-shell NTs	Electrochemical anodization	Ethanol/1930 ppm	S = (R_a_/R_g_) 0.8	100 °C/-	NA	[47]/2018
Al_2_O_2_/TiO_2_	Thermal oxidation	Ethanol/1000 ppm	S = (R_a_/R_g_) 1108.9	650 °C/-	4 min/20 min	[61]/2017
Nb_2_O_5_-TiO_2_ NFs	Electrospinning	Ethanol/500 ppm	S = (R_a_/R_g_) 21.64	250 °C0/45%	NA	[58]/2019
TiO_2_/V_2_O_5_ NFs	Electrospinning	Ethanol/100 ppm	S = (R_a_/R_g_) 24.6	350 °C/30%	6 s/7s	[74]/2016
Ni-TiO_2_ NTs	Electrochemical anodization	Acetone/1000 ppm	S = (∆R/R×100) 82%	100 °C/-	NA	[71]/2015
TiO_2_-SnO_2_ core-shell NFs	Electrospinning	Acetone/100 ppm	S = (R_a_/R_g_) 13.5	280 °C/-	2 s/60 s	[59]/2017
TiO_2_ NTs	Electrochemical anodization	Acetone/50 vol%	S = (∆R/R×100) 115%	Light irradiation at RT/-	NA	[68]/2020
TiO_2_ NWs	Vapor-Phase growth	CO/1 ppm	S = (NA) 11%	400 °C/-	NA	[63]/2016
TiO_2_ NRs	Hydrothermal	Ammonia/20 ppm	S = (∆R/R × 100) 14.1%	RT/50%	61 s/9 s	[44]/ 2020
Pbs QDs/TiO_2_ NTs	Electrochemical anodization	Ammonia/100 ppm	S = (R_a_/R_g_) 17.49	RT/-	NA	[70]/2016
TiO_2_ NTs	Electrochemical anodization	H_2_S/6 to38 ppm	S = (∆R/R × 100) 12 to 144%	70 °C/10%	NA	[72]/2016
Co-doped TiO_2_ NTs	Electrochemical anodization	H_2_S/50 ppm	S = (R_a_/R_g_) 199.16%	300 °C/50%	15 s/4 s	[73]/2019

**Table 5:** Summary of the gas sensing properties of TiO_2_-based nanostructure and heterostructure sensors towards oxidizing gases.

Material	Synthesis Method	Target Gas/ Concentration	Response (S)	Temperature/ Humidity	Response/ Recovery Time	Ref./Year
TiO_2_ NWs	Hydrothermal	NO_2_/100 ppm	S = (R_a_/R_g_) 3.1	RT/50%RH	10 s/19 s	[66]/(2018)
TiO_2_ NRs	Hydrothermal and annealing	NO_2_/40 ppm	S = (R_g_/R_a_) 1300	RT/dry air	NA	[64]/(2017)
TiO_2_-Al_2_O_3_ Core-shell NWs	Thermal oxidation	NO_2_/1000 ppm	S = (R_g_/R_a_) 1.9	650 °C/dry air	180 s/180 s	[61]/(2017)
MoS_2_-Decorated TiO_2_ NTs	Anodization and Hydrothermal	NO_2_/100 ppm	S = (R_g_/R_a_) 1.1	150 °C/45%RH	NA	[50]/(2016)
TiO_2_-In_2_O_3_ Composite NFs	Electrospinning	NO_2_/97 ppm	S = (∆R/R_a_) 95	RT/26%RH	6.7 s/NA	[60]/(2015)
TiO_2_ NTs	Anodization	NO_2_/50 ppm	S = (∆I/I_g_) 17	200 °C/50%RH	NA	[48]/(2018)
TiO_2_ Networked NWs	Vapor-phase growth	NO_2_/50 ppm	S = (∆R/R_a_×100) 8%	400 °C/dry air	NA	[63]/(2016)
TiO_2_-Ag_2_O Composite NRs	Hydrothermal and sputtering	NO_2_/0.5 ppm	S = (R_g_/R_a_) 3.1	250 °C/dry air	87 s/112 s	[65]/(2019)
TiO_2_@Au Heterojunction NRs	Hydrothermal and chemical approach	NO_2_/5 ppm	S = (∆R/R_a_) 135.5	250 °C/dry air	40 s/43 s	[94]/(2017)
TiO_2_ NRs Array	Acid vapor oxidation	O_2_/16% vol.	S = (R_g_/R_a_) 2.1	RT/dry air	55 s/51 s	[95]/(2016)
